# Dual-Source Contrast-Enhanced Multiphasic CT of the Liver Using Low Voltage (70 kVp): Feasibility of a Reduced Radiation Dose and a 50% of Contrast Dose

**DOI:** 10.3390/tomography9050125

**Published:** 2023-08-23

**Authors:** Keisuke Miyoshi, Masahiro Tanabe, Kenichiro Ihara, Masaya Tanabe, Mayumi Higashi, Koji Narikiyo, Yosuke Kawano, Atsuo Inoue, Katsuyoshi Ito

**Affiliations:** Department of Radiology, Yamaguchi University Graduate School of Medicine, Ube 755-8505, Japan

**Keywords:** CT, low-tube voltage, iodinated contrast media, radiation dose

## Abstract

This study investigated the feasibility of both a reduced radiation dose and a 50% of contrast dose in multiphasic CT of the liver with a 70 kVp protocol compared with a standard-tube-voltage protocol derived from dual-energy (DE) CT (blended DE protocol) with a full-dose contrast-agents in the same patient group. This study included 46 patients who underwent multiphasic contrast-enhanced dynamic CT of the liver with both a 70 kVp and a blended DE protocols. For quantitative analysis, median CT values for the liver, aorta, and portal vein, as well as signal-to-noise ratio (SNR) and contrast-to-noise ratio (CNR), were measured and calculated. In addition, as a qualitative analysis, the contrast effect and overall image quality of the abdominal organs were evaluated on a five-point scale. CNR and SNR of the hepatic parenchyma were not significantly different between the 70kV protocol and the Blended DE protocol in all phases. The 70 kVp protocol showed significantly better image quality compared with the blended DE protocol in the arterial phase (*p* = 0.035) and the equilibrium layer phase (*p* = 0.016). A 70 kVp CT protocol in combination with a reduced radiation dose and half-dose iodine load is feasible for multiphasic dynamic CT of the liver by maintaining the contrast enhancement effects and image quality in comparison with the blended DE CT protocol.

## 1. Introduction

Multiphasic contrast-enhanced dynamic computed tomography (CT) has been widely used for the detection and characterization of liver lesions as well as for the surveillance of chronic liver diseases [1]. Generally, this technique involves three-phase CT imaging, including arterial, portal, and equilibrium phases, in addition to precontrast CT. The clinical utility of this technique is, for instance, in the imaging-based diagnosis of HCC, which relies on hyperenhancement at the arterial phase and washout at the portal and/or equilibrium phases [2]. However, the acquisition of multiple phases by using CT is associated with a substantial increase in radiation dose compared with single-phase imaging, prompting concerns about an increased risk of radiation-induced carcinogenesis [3,4,5].

In addition, intravenous iodinated contrast material has been reported to be an important risk factor for nephrotoxicity (i.e., contrast medium-induced nephropathy [CIN]) for patients with chronic kidney diseases, although there are many other factors that contribute to the development of acute kidney injury [6,7]. Therefore, it is essential to reduce both the radiation dose and the amount of iodine contrast material delivered without reducing the image quality. 

In low-tube-voltage CT, increased iodine attenuation reduces the amount of iodine in the contrast agent while maintaining sufficient contrast resolution [8]. A previous study using high-tube-output dual-source CT showed that a low voltage (70 kVp) combined with a 50% iodine dose of contrast agent and an iterative reconstruction (IR) method had improved the contrast enhancement and image quality of abdominal organs compared with a conventional 120 kVp with a 100% iodine dose in multiphasic CT of the liver evaluated in the same patient group [9]. However, the limitations of this study were that the 70 kVp protocol used higher radiation dose (values represented by the CT dose index [CTDIvol]), compared with the 120 kVp protocol, since the automatic tube current control system based on the default setting was used; furthermore, the image reconstruction technique differed between IR in the 70 kVp protocol and filtered back projection (FBP) in the 120 kVp protocol. To resolve these limitations, an additional project was considered necessary, including the automatic adjustment of the tube current for dose reduction in the 70 kVp protocol and the application of the IR method instead of FBP in the 120 kVp protocol. Moreover, dual-energy (DE) CT has recently become widely used in clinical practice. Then, DE CT, in which a blend of low- and high-kVp images can be generated to provide an image impression similar to a standard 120 kVp image, was used for a standard tube voltage protocol (blended DE protocol) instead of the use of single-energy CT in this project.

The present study, therefore, assessed the feasibility of both a reduced radiation dose and a 50%-dose iodine load with the use of a low-concentrated contrast material in dual-source contrast-enhanced dynamic CT of the liver with a low voltage (70 kVp) with the IR method compared with a standard-tube-voltage protocol derived from DE CT (blended DE protocol) with the IR method and a full-dose iodine load in dual-source DE CT using the same group of adult patients.

## 2. Materials and Methods

### 2.1. Study Population

The institutional review board approval was obtained for this retrospective study, and the requirement for patient informed consent was waived. 

CT examinations that were performed in adults (>18 years old) from February 2019 to September 2021 using a dual-source CT system at a single large academic site were identified by querying the institutional radiology information system. Inclusion criteria were as follows: (a) multiphasic contrast-enhanced dynamic CT of the abdomen has been performed; (b) initial CT scans were obtained in 70 kVp dual-power mode with 50%-dose iodine using low-concentrated contrast agent; and (c) follow-up CT scans were obtained in dual-energy mode (tube voltage: 100 and 150 kVp) with a composition of the blended DE data set (corresponding to approximately 125 kVp: 50% from 100 and 150 kVp, respectively) and with full-dose iodine using medium-concentrated CM. 

We excluded from the analysis CT examinations of two patients for whom arterial phase images failed to be obtained. Finally, this study’s population included 46 patients (23 men, 23 women; age range, 49–89 years old; mean age, 75.6 years old) who met these criteria. The average patient’s body weight was under 73 kg (mean body weight, 54.2 ± 10.3 kg; range, 34–73 kg) based on our 70 kVp protocol. The time intervals between 70 kVp CT and blended DE CT were 188.9 ± 174.6 days (range: 32–917 days). The underlying diseases included chronic hepatitis or cirrhosis with periodic surveillance of hepatocellular carcinoma (*n* = 29), cancers of hepatobiliary (*n* = 7) and pancreatic (*n* = 7) origins, and pancreatic cystic lesions (*n* = 3).

### 2.2. CT

Abdominal CT in both protocols was conducted with a dual-source CT (SOMATOM FORCE; Siemens, Erlangen, Germany). This system has two X-ray tubes and two corresponding detectors set at an angle of about 95° and uses the automatic tube current modulation algorithm (Care Dose 4D; Siemens, Erlangen, Germany). 

In the 70 kVp protocol using a dual-power mode, CT scanning was performed in the craniocaudal direction from the top of the liver using the following parameters: detector collimation, 0.6 mm; tube rotation time, 0.5 s; pitch, 0.6; matrix, 512 × 512. Multiphasic contrast-enhanced dynamic CT consisted of precontrast, arterial phase (AP), portal venous phase (PVP), and equilibrium phase (EP). In this protocol, half the iodine dose (300 mgI/kg) of contrast agent compared with the blended DE protocol was used with a low-concentrated (140 mgI/mL) contrast agent (iohexol; Omniparque 140, GE HealthCare Pharma, Tokyo, Japan), and injected for 30 s using an automatic power injector. Immediately after, 30 mL of saline was flushed using the same injection rate. The delay time for AP scanning was individually determined using a bolus-tracking technique with the placement of a circular cursor on the abdominal aorta and the trigger threshold level set to the attenuation value of 100 Hounsfield units (HU), with a 20-second delay. The delay times for imaging in PVP and EP were 60 and 180 s after the administration of the contrast agent, respectively. Image reconstruction with a slice thickness and slice interval of 2 mm each was used, and an IR (Adaptative Model-based Iterative Reconstruction [ADMIRE]) level was set at 2. 

The blended DE protocol was performed using a dual-energy mode with a tube potential pair of 100 kVp and Sn 150 kV (where Sn denotes the interposition of a tin filter in the high-energy beam). Theoretically, the 70 kVp CT protocol allows a contrast reduction of about 51% compared to the 125 kVp CT protocol [10]. Therefore, using a blending factor of 0.5 (50% information of 100 kVp and 50% information of 150 kVp spectrum), linear blended images corresponding to the conventional CT acquisition at approximately 125 kVp were generated for the data analysis. The scan parameters were the same as the 70 kVp protocol. Multiphasic imaging was conducted with medium concentration (300 mgI/mL) of iodine contrast agent at full-dose (600 mgI/kg) (iohexol; Omniparque 300, GE HealthCare Pharma, Tokyo, Japan, or iopamidol; Iopamidol [F] 300, Fuji Pharma, Saitama, Japan) using a power injector. The injection techniques of contrast agent and image reconstruction (2-mm thickness and IR level of 2) in this blended DE protocol were also the same as in the 70 kVp protocol. Regarding the total volume of contrast agent, it was almost the same between the 70 kVp and blended DE protocols. For instance, a patient with a 50-kg body weight received a total of 107 and 100 mL of contrast agents, respectively. 

### 2.3. Image Analyses

All images were stored and reviewed on the dedicated workstation (EV InsiteS; PSP Corporation, Tokyo, Japan). Clinical information and scan parameters have been anonymized before being exported to the workstation for blinded data analysis. 

### 2.4. Quantitative Image Assessments

In the quantitative analyses, the CT values (HU) of the liver, abdominal aorta, portal vein, and spinal erector muscle were measured on axial images of two image sets by an experienced abdominal radiologist (K.M.) drawing circular regions of interest (ROIs) on the images. ROIs measurements were performed at the same location between different protocols at the level of the main portal vein. Care was taken to avoid focal lesions and visible vessels in the ROI measurements of the liver. Image noise (standard deviation [SD]) of the background air was also measured at the air space outside of the anterior abdominal wall. Then, the signal-to-noise ratio (SNR) and contrast-to-noise ratio (CNR) were calculated using the following respective equations: SNR = (CT value of interested area)/(SD of background air); CNR = (CT value of interested area—CT value of spinal erector muscle)/(SD of spinal erector muscle). The CTDIvol and dose length product (DLP) was also recorded in all phases to compare the radiation dose between the two protocols.

### 2.5. Qualitative Image Assessments

The qualitative analyses were performed by two independent radiologists (K.I., M.T.) with 6 and 5 years’ experience in abdominal CT reading who were blinded to the CT acquisition protocol. In cases with discrepancies in image interpretation, a consensus between readers was used to settle discrepancies. For the blind reading, the same soft-tissue window setting (width, 300 HU; level, 50 HU) was used in all examinations. Contrast enhancement of the organs, image noise, and the overall image quality were evaluated in all three phases for a comparison between the two protocols. In the AP, enhancement of the aorta, renal cortex, and pancreas as the representatives of hypervascular organs was assessed instead of the liver. In the PVP, the contrast effect of the liver parenchyma and portal vein was assessed, as was the contrast clarity between the hepatic parenchyma and portal vein. In the EP, the contrast effect of the hepatic parenchyma and hepatic veins was evaluated, as was the contrast clarity between them. 

Grading scores were recorded using the following 5-point scale based on the criteria used in the previous study [9]: enhancement of organs and overall image quality (1 = poor, 2 = suboptimal, 3 = average, 4 = good, 5 = excellent); clarity of contrast between the hepatic parenchyma and the portal/hepatic veins (1 = veins not visible, 2 = subtle, 3 = moderate, 4 = good, 5 = excellent); and image noise (1 = not diagnostic, 2 = severe, 3 = moderate, 4 = mild, 5 = absent). 

### 2.6. Statistical Analyses

Both quantitative and qualitative assessment scores between the 70 kVp and blended DE protocols were compared by using the Mann–Whitney U test. The interobserver agreement in qualitative parameters was compared by kappa statistical analyses (the kappa value of 0.81–1.00, excellent; 0.61–0.80, substantial; 0.41–0.60, moderate; 0.21–0.40, fair; <0.20, poor). 

All statistical analyses were performed using the IBM SPSS Statistics software program for Windows (Version 27.0., IBM Inc., New York, USA). Statistically significant *p*-values were set at <0.05. 

## 3. Results

In the comparison of the radiation dose between the two protocols, the median CTDIvol was significantly lower in the 70 kVp protocol than in the blended DE protocol in all three phases (AP: 5.79 vs. 8.35, *p* < 0.001, PVP: 7.37 vs. 8.66, *p* = 0.022, EP: 5.83 vs. 8.34, *p* < 0.001, respectively). 

**Figure 1:**
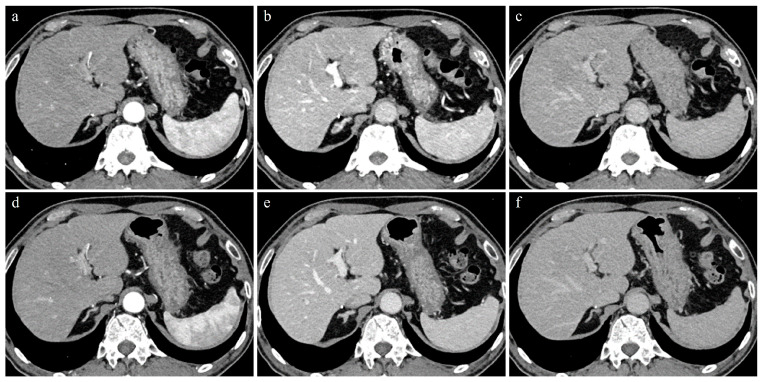
A 74-year-old man with 62.3kg body weight. (**a**–**c**) Initial CT images obtained with 70 kVp protocol during AP (**a**), PVP (**b**), and EP (**c**). (**d**–**f**) Follow-up CT images (9 months later) obtained with blended DE protocol during AP (**d**), PVP (**e**), and EP (**f**). All CT images were reconstructed by IR methods. Contrast enhancement of the hepatic parenchyma, portal vein, and hepatic vein in PVP and EP were better in 70 kVp than in blended DE protocol.

Table 1 shows the results of the comparison of quantitative image assessments between the two protocols. The median CT values (HU) of the hepatic parenchyma in the 70 kVp protocol were significantly higher than those in the blended DE protocol in all three phases (AP: 86.0 HU vs. 77.1 HU, *p* < 0.001; PVP: 119.8 HU vs. 108.0 HU, *p* < 0.001; EP: 101.9 HU vs. 91.4 HU, *p* < 0.001, respectively) (Figure 1). Conversely, median image noises (background SD) were significantly lower in the blended DE than in the 70 kVp protocol in the PVP (7.33 vs. 7.75, *p* = 0.002), while their differences were not statistically significant between the blended DE and 70 kVp protocols in the AP and EP (AP: 7.07 vs. 7.70, *p* = 0.118; EP: 7.19 vs. 7.33, *p* = 0.10, respectively). Regarding the CNR and the SNR, there were no significant differences between the 70 kVp and blended DE protocols in the CNR (AP: 3.5 vs. 2.8, *p* = 0.93; PVP: 7.3 vs. 6.4, *p* = 0.88; EP: 4.8 vs. 4.7, *p* = 0.65, respectively) or SNR (AP: 11.5 vs. 11.1, *p* = 0.44; PVP: 14.9 vs. 15.4, *p* = 0.39; EP: 13.7 vs. 13.3, *p* = 0.36, respectively) of the liver parenchyma in all three phases. 

The median CT values of the aorta in the AP and PVP were significantly higher in the 70 kVp than in the blended DE protocol (AP: 377.6 HU vs. 320.0 HU, *p* < 0.001; PVP: 181.4 HU vs. 153.7 HU, *p* < 0.001, respectively), while no significant differences were observed in the CNR and SNR of the aorta in the AP and PVP between the 70 kVp and blended DE protocols. In the EP, the median CT values, CNR, and SNR of the aorta were significantly higher in the 70 kVp protocol, compared with the blended DE protocol (135.6 HU vs. 111.2 HU, *p* < 0.001; 9.5 vs. 8.0, *p* = 0.001, 19.3 vs. 16.3, *p* = 0.019, respectively). 

The median CT values of the portal vein were significantly higher in the 70 kVp protocol than in the blended DE protocol in all three phases (AP: 162.2 HU vs. 126.1 HU, *p* < 0.001; PVP: 217.0 HU vs. 164.6 HU, *p* < 0.001; EP: 134.8 HU vs. 109.3 HU, *p* < 0.001, respectively). In addition, the CNR of the portal vein was significantly higher in the 70 kVp than in the blended DE protocol in the AP (13.8 vs. 10.4, *p* = 0.002) and EP (9.4 vs. 7.4, *p* = 0.002). The difference in the SNR of the portal vein was also significant between the 70 kVp protocol and the blended DE protocol in the AP (21.3 vs. 17.9, *p* = 0.002) and EP (SNR: 18.5 vs. 16.0, *p* = 0.017).

The results of qualitative analyses assessed by two radiologists are shown in Table 2. The inter-reader agreement was moderate to excellent for both protocols (kappa value: 0.41–1.00). Regarding the median scores of contrast enhancement of the organs, significantly higher scores were assigned in the 70 kVp than in the blended DE protocol in the pancreas (4.0 vs. 3.0, *p* < 0.001) and renal cortex (5.0 vs. 4.0, *p* < 0.001) in the AP, in the portal vein (5.0 vs. 4.0, *p* < 0.001) in the PVP, and in the hepatic parenchyma (3.0 vs. 3.0, *p* < 0.001) and hepatic vein (3.0 vs. 2.0, *p* = 0.001) in the EP. 

Regarding the overall image quality, it was significantly better in the 70 kVp than in the blended DE protocol in the AP (4.0 vs. 4.0, *p* = 0.035) and EP (3.0 vs. 3.0, *p* = 0.016) (Figure 1). Conversely, the median image noise scores were significantly better in the blended DE than in the 70 kVp protocol in all phases (AP: 4.0 vs. 3.5, *p* < 0.001, PVP: 4.0 vs. 4.0, *p* = 0.046, EP: 4.0 vs. 3.0, *p* = 0.004, respectively). 

In the comparison in clarity of contrast between the hepatic parenchyma and portal vein in the PVP and between the hepatic parenchyma and hepatic vein in the EP, the median scores in the 70 kVp protocol were significantly higher than those in the blended DE protocol (5.0 vs. 4.0, *p* < 0.001; 3.0 vs. 2.0, *p* < 0.001, respectively) (Figure 1). 

## 4. Discussion

Low-tube-voltage CT is known to be an effective technique for reducing the CM iodine dose because the increased mass attenuation coefficient of iodine improves the effectiveness of the contrast agent [11,12,13]. The previous study also showed that using the 70 kVp protocol can reduce the amount of CM iodine required for adequate enhancement of the liver parenchyma by 50% [9]. However, there were two limitations in that study: the use of an increased radiation dose to compensate for the increased image noise compared with the 120 kVp protocol and the differences in the image reconstruction methods between the 70 kVp protocol using IR and the 120 kVp protocol using FBP. 

The findings of the present study designed to resolve these issues revealed that the image quality, as well as the enhancement effect of the hepatic parenchyma and the portal vein in the 70 kVp protocol with reduced radiation dose and a half-dose iodine load, were comparable to or better than those in the standard-tube-voltage (blended DE) protocol with a full-dose iodine load, under the same IR algorithm in the same patient group. This fact suggested that the reduced iodine dose of 50% still preserved satisfactory hepatic enhancement and the image quality even with a reduced radiation dose, indicating the feasibility of the 70 kVp protocol with reduced iodine and radiation doses for contrast-enhanced dynamic CT of the liver in clinical practice under the use of the dual-power mode of high tube output dual-source CT. Especially in patients with chronic liver disease who have risk factors for renal injury, such as diabetes [14], a 70 kVp protocol with reduced iodine and radiation doses is desirable to reduce the risk of renal failure. 

Low-tube-voltage CT with reduced radiation dose has some disadvantages, such as increased image noise and the consequent deterioration of the image quality [5]. The use of a high tube current output and IR (ADMIRE) algorithm in this 70 kVp protocol was suggested to have maintained the overall image quality at a level similar to that with the blended DE protocol. In contrast-enhanced liver CT, effective contrast enhancement of the liver parenchyma is known to be essential for the visualization of focal hepatic lesions, especially in the PVP and EP. The 70 kVp protocol with reduced iodine and radiation doses in this study showed better CT values of the liver parenchyma than the blended DE protocol in all three phases, similar to the results of the previous study [9]; this was probably because the CT attenuation value was less markedly affected by the reduced radiation dose.

Regarding the reduction in the iodine dose of CM (half dose = 300 mgI/kg), the advantage of using low-concentration CM (140 mgI/mL) in this study was that the established routine injection protocol, such as the standard-tube-voltage protocol, could be applied to the 70 kVp protocol since the total volume of injected CM was almost the same as with blended DE protocol. If we had used CM with medium- or high-concentration (300 or 370 mgI/mL) to reduce the total iodine dose, the injection protocol would need to have been changed since the total injection volume of CM would also have been reduced. In addition, changing the injection protocol might affect the detection of hepatic lesions, especially hypervascular nodules, such as hepatocellular carcinoma, due to deviations in the optimal timing of the arterial phase. 

Several limitations associated with our study merit consideration. First, the number of subjects was small. As we evaluated the differences in the contrast enhancement effects and image qualities between the low- and standard-tube-voltage protocols, only relatively thin patients (<73 kg of body weight due to clinical restrictions) who underwent both protocols were included, which limited the number of included patients. The small sample size limited the generalization of our results. Second, the 70 kVp protocol was applied exclusively to patients <73 kg of body weight in this study. Therefore, the mean body weight of our patients was 54.2 kg, which is smaller than that of the general European and North American populations, so the results may differ for larger or obese patients, even in Asian populations, which tend to have lean body types. In fact, several previous studies have shown that increased image noise of low-voltage CT is problematic in heavier patients [5,15,16,17], so low-tube-voltage CT is not as appropriate as standard-tube-voltage CT for these patients [6,18]. Further studies are needed to modify the low-tube-voltage CT protocol and render it applicable to patients with higher body weights. Third, this study did not evaluate whether or not the low-tube-voltage CT protocol with reduced iodine and radiation doses affected the diagnostic performance for lesion detection of the liver since the primary purpose of this study was to assess the contrast enhancement effects and image quality. This point should be clarified in future studies. Furthermore, the optimal settings of low-tube-voltage CT need to be determined for hepatic dynamic CT for the detection and characterization of focal hepatic lesions. Finally, the measurements of the CT values and image noise were performed by only one reader, so we were unable to evaluate the intra- or inter-reader variations for quantitative data. 

## 5. Conclusions

A 70 kVp combined with a reduced radiation dose and half-dose iodine using a low-concentration contrast agent in dual-source CT is feasible for multiphasic (AP, PVP, and EP) dynamic CT of the liver in lean patients by maintaining the contrast enhancement effects and image quality in comparison with the blended DE protocol using dual-source dual-energy CT.

## Figures and Tables

**Table 1:** Quantitative image assessment in the comparison between 70 kVp and blended DE protocol.

		70 kVp	Blended DE	*p*-Value
Arterial phase				
Liver	CT value	86.02 (14.73)	77.11 (13.82)	<0.001
	SNR	11.53 (4.48)	11.10 (2.90)	0.435
	CNR	3.45 (3.61)	2.82 (2.51)	0.926
Aorta	CT value	377.56 (83.45)	319.96 (89.46)	<0.001
	SNR	54.53 (15.58)	48.87 (15.15)	0.076
	CNR	47.37 (14.70)	40.38 (16.93)	0.052
PV	CT value	162.24 (51.11)	126.10 (47.48)	<0.001
	SNR	21.26 (8.07)	17.87 (9.22)	0.002
	CNR	13.78 (6.55)	10.44 (7.60)	0.002
BG	SD	7.70 (1.98)	7.07 (1.85)	0.118
CTDIvol (mGy)		5.79 (3.07)	8.35 (2.71)	<0.001
DLP (mGy·cm)		163.6 (101.97)	234.05 (91.75)	0.023
Portal venous phase				
Liver	CT value	119.78 (19.87)	108.03 (22.26)	<0.001
	SNR	14.89 (6.35)	15.41 (5.47)	0.385
	CNR	7.31 (5.76)	6.36 (3.87)	0.883
Aorta	CT value	181.34 (28.08)	153.66 (22.70)	<0.001
	SNR	24.20 (6.65)	23.03 (10.33)	0.674
	CNR	15.15 (5.52)	13.93 (8.17)	0.181
PV	CT value	217.00 (53.38)	164.55 (31.38)	<0.001
	SNR	28.09 (11.01)	24.30 (12.01)	0.240
	CNR	19.81 (9.62)	15.97 (9.52)	0.051
BG	SD	7.75 (2.20)	7.33 (2.44)	0.002
CTDIvol (mGy)		7.37 (4.18)	8.66 (2.75)	0.022
DLP (mGy·cm)		465.1 (331.32)	467.65 (254.67)	0.523
Equilibrium phase				
Liver	CT value	101.91 (9.79)	91.42 (12.13)	<0.001
	SNR	13.74 (4.64)	13.29 (4.14)	0.362
	CNR	4.76 (3.29)	4.66 (2.04)	0.650
Aorta	CT value	135.61 (20.00)	111.21 (18.14)	<0.001
	SNR	19.25 (4.77)	16.27 (5.48)	0.019
	CNR	9.46 (4.05)	7.99 (3.45)	0.001
PV	CT value	134.78 (17.93)	109.31 (19.7)	<0.001
	SNR	18.48 (5.23)	15.98 (5.52)	0.017
	CNR	9.41 (3.77)	7.41 (3.89)	0.002
BG	SD	7.33 (2.10)	7.19 (1.78)	0.100
CTDIvol (mGy)		5.83 (3.05)	8.34 (2.63)	<0.001
DLP (mGy·cm)		162.2 (101.22)	232.75 (91.07)	0.002

Note—Data are the medians (interquartile ranges); PV = portal vein; BG = background; SD = standard deviation; CNR = contrast-to-noise ratio; SNR = signal-to-noise ratio; CTDIvol = volume CT dose index; DLP = dose length product.

**Table 2:** Qualitative image assessment (median scores) between 70 kVp and blended DE protocol.

	70 kVp	Blended DE	*p*-Value
Arterial phase			
Contrast enhancement			
Aorta	5.0 (0)	5.0 (0)	N.S.
Renal cortex	5.0 (1)	4.0 (1)	<0.001 *
Pancreas	4.0 (1)	3.0 (1)	<0.001 *
Image noise	3.5 (1)	4.0 (0)	<0.001 ^†^
Overall image quality	4.0 (0)	4.0 (1)	0.035 *
Portal venous phase			
Contrast enhancement			
Liver	4.0 (0)	4.0 (1)	0.093
PV	5.0 (1)	4.0 (0)	<0.001 *
Contrast between PV and liver	5.0 (1)	4.0 (0)	<0.001 *
Image noise	4.0 (1)	4.0 (0)	0.046 ^†^
Overall image quality	4.0 (0)	4.0 (0)	0.527
Equilibrium phase			
Contrast enhancement			
Liver	3.0 (0)	3.0 (0)	<0.001 *
Hepatic vein (HV)	3.0 (2)	2.0 (1)	0.001 *
Contrast between HV and liver	3.0 (2)	2.0 (1)	<0.001 *
Image noise	3.0 (0)	4.0 (1)	0.004 ^†^
Overall image quality	3.0 (1)	3.0 (0)	0.016 *

Note—Data are the medians (interquartile ranges). HV = hepatic vein. * The score for 70 kVp is statistically higher than that for blended DE. ^†^ The score for blended DE is statistically higher than that for 70 kVp.

## Data Availability

Not applicable.
